# Trend of microbiologically-confirmed tuberculosis in a low-incidence setting with high immigration rates

**DOI:** 10.1186/1471-2458-14-340

**Published:** 2014-04-10

**Authors:** Giulia Lombardi, Paola Dal Monte, Agnese Denicolò, Marina Tadolini, Giulia Martelli, Maria Letizia Bacchi Reggiani, Pierluigi Viale, Maria Paola Landini

**Affiliations:** 1 Microbiology Unit - Department of Experimental, Diagnostic and Specialty Medicine, Alma Mater Studiorum University of Bologna, S. Orsola-Malpighi University Hospital, Via Giuseppe Massarenti 9, 40138 Bologna, Italy; 2 Infectious Diseases Unit - Department of Medical and Surgical Sciences, Alma Mater Studiorum University of Bologna, S. Orsola-Malpighi University Hospital, Via Giuseppe Massarenti 9, 40138 Bologna, Italy; 3 Cardiology Unit - Department of Experimental, Diagnostic and Specialty Medicine, Alma Mater Studiorum University of Bologna, S. Orsola-Malpighi University Hospital, Via Giuseppe Massarenti 9, 40138 Bologna, Italy

**Keywords:** Tuberculosis, Epidemiology, Immigration, Drug resistance, Microbiological diagnosis

## Abstract

**Background:**

The metropolitan area of Bologna, a city in Northern Italy (Emilia Romagna region), is considered a low incidence setting for TB, but has a high rate of foreign immigration (13.5% official resident immigrants relative to the whole population in 2011). The aim of this study was to describe the epidemiological trend of TB, focusing on differences between Italian and foreign-born cases.

**Methods:**

We examined all bacteriologically confirmed TB cases identified in the Microbiology Unit of Bologna University Hospital from January 2008 and December 2011. We compared demographic, clinical and microbiological data for Italian vs. foreign-born TB cases.

**Results:**

Out of 255 TB cases identified during the study period, 168 (65.9%) were represented by foreign-born cases. The proportion of immigrants with TB progressively increased over the study period (from 60.8% in 2008 to 67.5% in 2011). Although foreign-born cases were significantly younger than Italian cases (mean age 32.3 ± 14.4 years vs 61.9 ± 21.5 years), the mean age among the latter decreased from 71.2 in 2008 to 54.6 years in 2011 (p = 0.036).

Concerning TB localization, 65.9% (n = 168) had pulmonary TB (P-TB) and 34.1% (n = 87) extra-pulmonary TB (EP-TB). In this study, 35.6% of Italian-born P-TB cases were smear positive, versus 51.4% of foreign-born P-TB cases. The highest proportion of high-grade positive microscopy P-TB was among subjects between 25–34 years old (36.9%; p = 0.004).

Mono-resistance to isoniazid (mono-H) was found among 9.2% and 10.1% of Italian and foreign-born cases, respectively. Among Italian cases, resistance to H and any other first line drug (poly-H) and Multidrug resistant TB (MDR-TB) were 4.6% and 1.2%, respectively. In foreign-born cases poly-H (12.8%) and MDR-TB (6.9%) significantly increased over the time (p = 0.003 and p = 0.007, respectively). The proportion of MDR-TB was significantly higher among immigrants from Eastern Europe (10.9%) compared to Italian-born patients (p = 0.043). All (n = 9) MTB strains resistant to four or five first line drugs and Extensively drug resistant (XDR-TB) strains were from foreign-born cases.

**Conclusions:**

TB epidemiology in a low incidence setting is strongly influenced by immigration rates. Ethnicity, mean age, and incidence of MDR-TB among foreign-born cases reflect immigration trends in Northern Italy.

## Background

Tuberculosis (TB) in industrialized countries has re-emerged as a public health concern after decreases in incidence during the 20th century. Despite nation-specific quantitative changes (decrease, stabilization, or increase) in overall TB notification rates for past 30 years, a similar underlying trend in TB epidemiology has emerged in industrialized countries: a decreasing incidence in the native population and an increasing incidence in foreign-born persons [1,2].

In 2012 the World Health Organization estimated 8.6 million incident cases of TB worldwide [3]. Although the incidence of TB steadily decreased in most wealthy Western European and Northern American countries in the last few decades, the proportion of cases in immigrants from high-burden countries has increased. Currently a large proportion of all new TB cases (>50%), occurs among foreign-born people [4,5].

In line with this trend, the incidence of newly reported TB cases in Italy has remained relatively stable over the last decade at 7 cases per 100,000 people per year, but the proportion of foreign-born TB cases increased from 22% in 1999 to 46% in 2008 [6]. This trend corresponds to doubling of foreign-born population during the same timeperiod [7].

Recently published data examining the epidemiology of TB in the Italian region of Emilia-Romagna from 1996–2011, identified a trend of increasing TB cases among immigrants from 19.1% in 1996 to 53.3% in 2006 [8], and to 59.6% in 2011 [9]. In the same timeperiod, the proportion of resident immigrants (subjects who hold an official residence permit) relative to the whole population increased from 1.1% in 1993 to 11.9% in 2011 [10]. In 2011, Emilia Romagna region accounted for 437 new cases of tuberculosis out of a total population of 4.35 million inhabitants, corresponding to a notification rate of 10.7 TB cases/100,000 inhabitants [9].

The aim of this study was to analyze the demographic, clinical and microbiological characteristics of bacteriologically-confirmed TB cases of the Bologna metropolitan area, setting with low TB incidence and high immigration rate, between 2008 and 2011, and to describe the epidemiological trend of TB, focusing on differences between Italian and foreign-born cases.

## Methods

### Setting

This observational retrospective study was conducted at the Microbiology Unit of S. Orsola-Malpighi University Hospital of Bologna. Bologna is considered a low incidence setting for TB (from 15.4 cases/100,000 inhabitants in 2008 to 12.1 cases/100,000 inhabitants in 2011) [9,11,12]), but has a high rate of foreign immigration (from 10.5% official resident immigrants relative to the whole population in 2008 to 13.5% in 2011) [13]. The Microbiology Unit is one of the two referral centres for the diagnosis of TB serving the metropolitan area of Bologna, with a total population of approximately 853,291 inhabitants in 2011 [9].

All bacteriologically confirmed TB cases (n = 255) identified in our Unit between January 2008 and December 2011 were included in the study. A total of 474 notified TB cases have been reported in Bologna during the same time period [9,11,12]; out of them 28.3% (n = 134) were not culture confirmed. Therefore our study accounts for 53.8% of the total TB cases in Bologna, and 75.0% of culture-confirmed cases, making the data representative of disease trends of the metropolitan area.

Bacteriologically-confirmed TB was defined by any positive culture for *Mycobacterium tuberculosis* (MTB). For each TB case, we collected data on the patient demographic (age, gender, country of origin, ethnicity), clinical (pulmonary and/or extra-pulmonary TB localization) and microbiological (e.g. smear microscopy results, susceptibility to First Line Drugs-FLD) characteristics. The protocol for this study was reviewed and approved by the Ethics Committee of the S.Orsola-Malpighi University Hospital (Prot. n. 3229/2013).

### Microbiological diagnosis

All specimens were stained for acid-fast microscopic examination using Ziehl-Neelsen stain. The grade of acid-fast bacilli positivity was recorded according to five categories (scanty, 1+, 2+, 3+ and 4+) according to published guidelines [14,15]. The molecular GeneXpert MTB/RIF (Cepheid, USA) assay was performed for confirmation.

MTB was isolated using solid (Lowenstein-Jensen; Heipha Diagnostika Biotest, Germany) and liquid culture (MGIT 960; Becton Dickinson, USA). MTB culture identity was confirmed with MGIT TBc Identification Test (Becton Dickinson, USA). Drug susceptibility test (DST) to first-line drugs (FLDs) streptomycin (S), isoniazid (H), rifampicin (R), ethambutol (E) and pyrazinamide (Z), was performed by the “gold standard” automatic MGIT 960 system (Becton Dickinson) at the following concentrations 1.0, 0.1, 1.0, 5.0 and 100 μg/ml. To confirm resistance, DST was repeated, using two different drug concentrations (1.0 and 4.0 for S, 0.1 and 0.4 for H, 5.0 and 7.5 μg/ml for E).

In case of a multidrug-resistant (MDR) strain, defined as resistant to at least R and H, a DNA strip assay (Genotype MTBDR-sl, Hain Lifescience, Germany) was used to detect resistance pattern to aminoglycosides/cyclic peptides and fluoroquinolones, while phenotypic susceptibility to second line drugs was tested in the National MDR-TB Reference centre of Sondalo (Sondrio, Italy). Extensively drug-resistant (XDR) isolates were defined as MDR-TB also resistant to any of the fluoroquinolones and at least to one of three injectable second-line drugs, according to WHO definitions [16].

### Statistical analysis

Study variables were analyzed comparing Italian and foreign-born cases. Continuous variables (i.e. age) were compared using the Student t-test for unpaired data. Annual trends of cases, mean age and resistances among Italian and foreign-born were investigated through linear regression models and estimate of unstandardized coefficients (B) and their confidence intervals (95% CI) are reported. Categorical data were analysed by χ2 test. Two-way analysis of variance was performed to analyse the age of Italian and foreign-born patients during the period 2008–2011. A p value less than 0.05 was considered statistically significant. Statistical analysis was performed using the statistical software Stata/SE 12.1 (StataCorp LP, College Station, USA).

## Results

Two hundred and fifty-five (255) MTB strains were isolated between 2008 and 2011, using standard microbiological procedures. Of these isolates, 65.9% (n = 168) were from foreign-born patients while 34.1% (n = 87) were Italian-born patients. MTB and HIV co-infection accounted for 11 out of 76 cases tested.

The annual proportion of foreign-born cases out of the total MTB isolation increased from 60.8% in 2008 to 67.5% in 2011. Linear regression confirmed a significant increasing trend in the number of foreign-born cases (B = 7.6; 95% CI 1.95–13.24; p = 0.030).

Emigrants from Asia represented the largest proportion of foreign-born cases (n = 66, 39.3%), followed by Eastern Europeans (n = 49, 29.2%), Africans (n = 45, 26.8%) and South Americans (n = 8, 4.7%). Among Asian populations, most patients had emigrated from Pakistan (48.5%). Romanian emigrants accounted for 69.3% of Eastern-European TB cases, whereas Morocco accounted for 33% of African MTB cases. If analysed by country of origin, the majority of TB cases among foreigners were from Romania (20.2%) and Pakistan (19.0%), followed by Morocco (8.9%).

A majority of TB cases were identified in males (n = 156, 61.2%). No differences in the sex-distribution of TB cases was observed between Italian-born and foreign-born TB cases, with the exception of Eastern European emigrants, where females represented the majority (57.1%) of cases (p = 0.006).

The mean age for Italian-born TB cases was 61.9 years (±21.5 SD), whereas foreign-born TB cases were significantly younger (mean age of 32.3 ± 14.4 years; p = 0.0001). However, the mean age among Italian-born TB cases decreased from mean age of 71.2 years in 2008 to 54.6 years in 2011 (B = −4.2; 95% CI −8.2-0.3; p = 0.036). Among foreign-born cases, 76% was between 15 and 44 years old while 75% of Italian-born cases was older than 45 years old (p = 0.0001).

Concerning TB localization, 65.9% (n = 168) had pulmonary TB (P-TB) and 34.1% (n = 87) extra-pulmonary TB (EP-TB). Common extra-pulmonary sites included peripheral lymph nodes (35.6%), pleura (18.4%), osseous (9.2%), urinary tract (8%) and meningeal (4.6%) localization. According to ethnicity, significantly different patterns of TB localization were found among Eastern European patients (81.6% P-TB vs 18.4% EP-TB) and Africans (48.9% P-TB vs 51.1% EP-TB) (p = 0.017).

From an epidemiological standpoint, pulmonary TB sputum smear positive is the greatest concern because of the high risk for transmission among contacts. Smear positive microscopy samples accounted for 45.8% (n = 77) of P-TB cases. In our analysis, 35.6% of Italian-born P-TB cases were smear positive, against 51.4% of foreign-born P-TB cases. With reference to sputum smear microscopy grading, there was a higher proportion of high-grade (3+, 4+) smear-positive P-TB among foreign-born (26.6%) than Italian-born (11.9%) cases (p = 0.059). Stratification by age showed that the highest frequency of high-grade smear positivity was in the age band 25–34 years (36.9%; p = 0.004).

**Figure 1:**
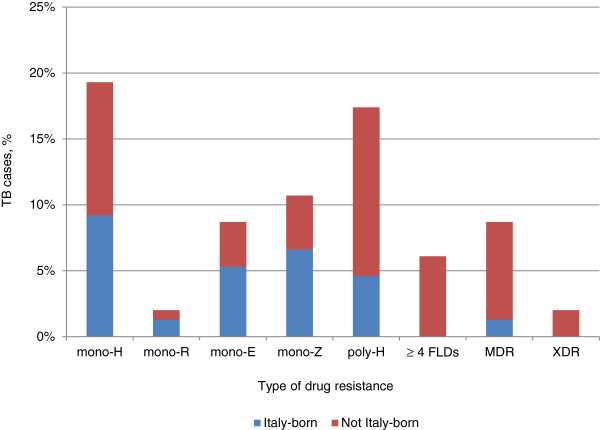
Rate of anti-TB drugs resistance among Italian-born and foreign-born people.

Anti-TB drugs susceptibility data were available in 239 (93.7%) out of 255 strains. Figure 1 depicts the distribution of respective resistance patterns in Italian vs. foreign-born TB cases. Specifically, mono-resistance to isoniazid (mono-H) in Italian-born cases was documented in 9.2% of cases, with resistance to H and any other FLD (poly-H) in 4.6% of cases and MDR-TB in 1.3% of cases. Among foreign-born TB cases, mono-H resistance was found in 10.1%, poly- H in 12.8% of cases, and MDR-TB in 7.4% of cases. The proportion of MDR-TB was significantly higher among immigrants from Eastern Europe versus Italian-born (10.9% vs. 1.3%; p = 0.043). All (n = 9) MTB strains resistant to four or five FLDs and XDR strains were from foreign-born cases.

**Figure 2:**
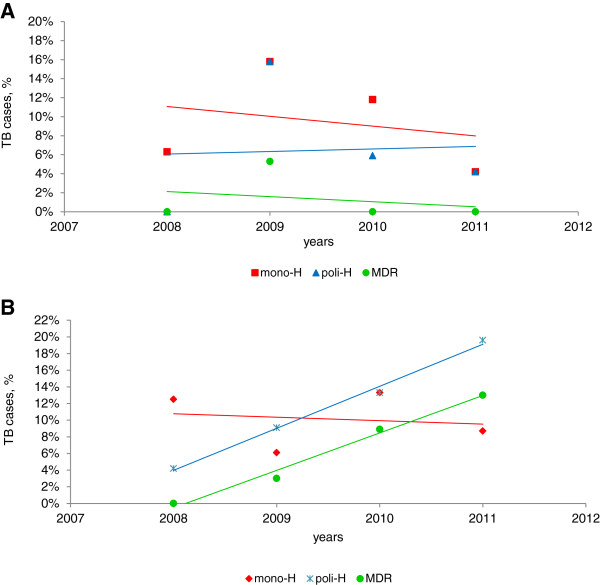
Trends of mono-H, poly-H and MDR-TB cases among Italian-born (A) and foreign-born (B) people during the period 2008–2011.

Figure 2 describes the annual proportion of mono-H, poly-H and MDR in Italian vs. foreign-born TB cases. Resistance trends were relatively stable among Italians, while among foreign-born cases poly-H and MDR increased significantly over time: poly-H from 4.2% in 2008 to 19.6% in 2011 (B = 5.04; 95% CI 3.76-6.31; p = 0.003) and MDR from 0.0% in 2008 to 13.0% in 2011 (B = 4.49; 95% CI 2.87-6.10; p = 0.007).

## Discussion and conclusions

Emigration patterns are increasingly influencing the epidemiology of tuberculosis in low-incidence Western countries. Patients from regions with relatively high prevalence rates of latent TB can reactive their infection, in some cases caused by MDR strains, years later after arrival to a low-incidence region. This study described the epidemiological trend of TB in the metropolitan area of Bologna, a low TB incidence setting of Northern Italy with a high immigration rate, during the period 2008–2011.

In line with last surveillance reports published by Emilia-Romagna Region [9,11,12], our study confirmed an increasing percentage of TB cases among emigrant patients that reached 67.5% of total confirmed TB cases in 2011. In particular, the majority of the cases were from Romania and Pakistan, followed by Morocco. These data contrast with previous findings reported by Odone et al. who described the trend of TB in Emilia-Romagna during the period 1996–2006, when most TB cases were from Morocco [8]. Our results may reflect a change in the immigration flux in Bologna province during recent years. In addition, the finding that Eastern European emigrants with TB were mainly females reflects a social trend of predominantly female emigration to our area from Eastern Europe.

As previously reported, foreign-born TB cases were significantly younger than Italian-born cases, even though, interestingly, the mean age of the latter is decreasing. The decreasing age among Italians with TB could be explained by higher proportion than in the past of young people affected by immune disorders, resulting in a reduced control of tuberculosis infection. Future studies could confirm this observation.

We observed important differences in the clinical manifestation of TB among Italian and foreign-born patients. In particular among Africans, EP-TB were more frequent that P-TB, while among East-European born people the vast majority of cases were P-TB. However, the association between TB localization and ethnicity may be the result of confounders requiring multivariable analysis.

Grading of sputum smear microscopy results is rarely reported in literature, but it could predict the risk of transmission by case and the speed of bacteriologic conversion during chemotherapy [17]. Foreign-born cases showed a higher frequency of high-grade smear-positive P-TB than Italian-born, possibly due to late access to medical examination, when the disease is in a more advanced clinical stage.

A major issue in the global control of TB is the rise of drug resistant strains, because their treatment is difficult, expensive, prolonged and associated with serious side-effects. A retrospective study conducted by Fattorini and colleagues about drug resistant TB in Italy over the period 2008–2010 reported an overall MDR-TB rate of 3.8%, with large differences in MDR-TB rate among Italians (1.4%), immigrants from Former Soviet Unit (30.3%) and from Romania (5.9%) [18]. In our study the overall MDR-TB proportion was 5.0% with the highest rate among Eastern European-born persons (10.9%), compared with Asians (4.7%), Africans (2.4%) and Italians (1.3%).

With respect to multi-drug resistance, all strains found to be resistant to four or five FLDs and XDR strains were identified exclusively foreign-born patients. Furthermore, we observed a significant increase in poly-H resistant and MDR strains during the study period in foreign-born TB cases.

In conclusion, the present data show a progressive increase in the proportion of TB cases in foreign-born subjects in the Bologna area over time. In addition to the clear increase of immigration in the area, this finding might suggest the need to intensify the screening of immigrants for TB and latent tuberculosis infection, and make the TB prevention, diagnosis and treatment service more accessible to the immigrant population.

## Competing interests

The authors declare that they have no competing interest.

## Authors’ contributions

GL, PDM, AD participated in the conception and study design, performed microbiological diagnosis of TB, managed microbiological data with their analysis and interpretation and drafted the article. MT and GM acquired data on patients admitted to the Infectious Disease Unit and participated in their analysis and interpretation. MLBR performed statistical analysis. PV and MPL participated in the critical revision of the study and gave final approval to the version to be published. All authors read and approved the final manuscript.

## Pre-publication history

The pre-publication history for this paper can be accessed here:

http://www.biomedcentral.com/1471-2458/14/340/prepub
